# Challenges and Economic Implications in the Control of Foot and Mouth Disease in Sub-Saharan Africa: Lessons from the Zambian Experience

**DOI:** 10.1155/2014/373921

**Published:** 2014-08-21

**Authors:** Y. Sinkala, M. Simuunza, D. U. Pfeiffer, H. M. Munang'andu, M. Mulumba, C. J. Kasanga, J. B. Muma, A. S. Mweene

**Affiliations:** ^1^Department of Veterinary Services, Ministry of Agriculture and Livestock, P.O. Box 50060, Lusaka, Zambia; ^2^Department of Disease Control, School of Veterinary Medicine, University of Zambia, Lusaka, Zambia; ^3^Royal Veterinary College, London AL9 7TA, UK; ^4^Norwegian School of Veterinary Sciences, Ullevålsveien, P.O. Box 8146, 0033 Oslo, Norway; ^5^Onderstepoort Veterinary Institute, Agricultural Research Council, Old Soutpan Road, Onderstepoort, Pretoria 0110, South Africa; ^6^Sokoine University of Agriculture, P.O. Box 3297, Chuo Kikuuu, Morogoro, Tanzania

## Abstract

Foot and mouth disease is one of the world's most important livestock diseases for trade. FMD infections are complex in nature and there are many epidemiological factors needing clarification. Key questions relate to the control challenges and economic impact of the disease for resource-poor FMD endemic countries like Zambia. A review of the control challenges and economic impact of FMD outbreaks in Zambia was made. Information was collected from peer-reviewed journals articles, conference proceedings, unpublished scientific reports, and personal communication with scientists and personal field experiences. The challenges of controlling FMD using mainly vaccination and movement control are discussed. Impacts include losses in income of over US$ 1.6 billion from exports of beef and sable antelopes and an annual cost of over US$ 2.7 million on preventive measures. Further impacts included unquantified losses in production and low investment in agriculture resulting in slow economic growth. FMD persistence may be a result of inadequate epidemiological understanding of the disease and ineffectiveness of the control measures that are being applied. The identified gaps may be considered in the annual appraisal of the FMD national control strategy in order to advance on the progressive control pathway.

## 1. Introduction

Zambia is a sub-Saharan African country with potential to boost its economy through trade in livestock commodities [1, 2]. However, this potential is hindered by the presence of numerous disease challenges including foot and mouth disease (FMD). FMD is an endemic disease in Zambia that hinders economic exploitation of the livestock resource in a country where 80% of the population is dependent on agriculture [1, 3, 4]. The disease is described as an acute, highly contagious viral infection of wild cloven-hoofed animals involving more than 70 wildlife species and domesticated ruminants including pigs [5]. The disease is caused by FMD virus (FMDV), which is a prototypic member of the genus* Aphthovirus*. There are seven FMDV serotypes: A, O, C, SAT 1, SAT 2, SAT 3, and Asia 1 [6]. In southern Africa, two distinct molecular epidemiological forms of the disease exist [7], which are the Southern African Territories (SAT) serotypes and the Eurasian or South American (A and O) serotypes.

FMD has persisted in Zambia since the first recorded outbreak in 1933 [8]. The persistence may be a result of inadequate epidemiological understanding and ineffective control measures [9]. Other reasons include the presence of the reservoir African wild buffalo (*Syncerus caffer*) [10]; presence of five serotypes O, A, SAT 1, 2, and 3; the virus' ability to persist in the environment without animal hosts; and lack of a definitive domestic host for life cycle [11]. The Food and Agriculture Organization (FAO) of the United Nations (UN) FMD progressive control pathway (PCP) classified Zambia as being at level 2 [12]. And as such, identification of FMD control challenges and economic impacts may provide opportunities for investment in advancing to the desired level 3 classification of the (Organization of International Epizootics (OIE) progression criteria. To attain this, it is imperative that we understand the limitations that have led Zambia not to progress into attaining the desired level 3 criteria. Therefore, the objective of this review was to describe the control challenges and economic impacts of FMD outbreaks in Zambia. In doing so, we reviewed published literature on FMD outbreaks, control, and impact. The literature search was conducted on major electronic databases including PubMed, Science Direct, Cabi direct, and Springer using specified search terms. The search terms included “aphthous fever, aphthae epizooticae, hoof and mouth disease, foot and mouth disease, and Zambia.” The inclusion criteria were studies published in English in peer-reviewed journals and studies where full text was available. Further, grey literature from government institutions was obtained. These included annual reports from the Ministry of Agriculture and Livestock for the period 1933 to 1912, economic and policy documents from the Ministry of Finance and National Planning as well as the Bank of Zambia between 2000 and 2010, and Central Statistical Office population census reports 2000 and 2010. Furthermore, expert opinions on impacts of FMD were obtained through focused discussions with farmers, scientists, commodity associations, and personal field experiences. We discuss the findings with the aim of bringing into perspective the challenges experienced by the government in controlling FMD and the economical implication of the disease in Zambia that would serve as an example to other countries faced with similar challenges. And we also highlight the possible solution that would help overcome these predicaments.

## 2. Challenges in the Livestock Production System

**Figure 1:**
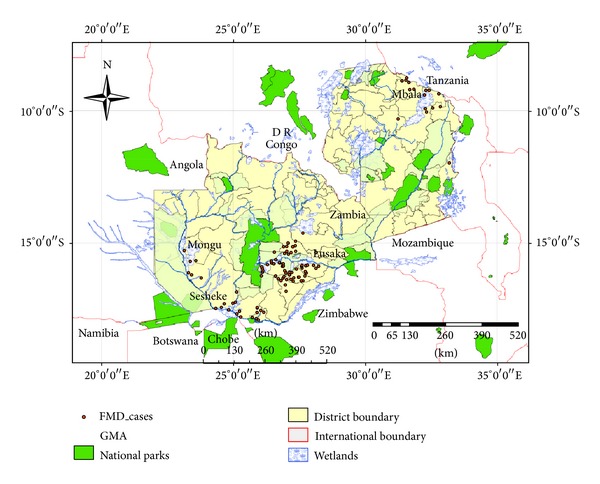
Showing distribution of FMD cases in relation to wildlife protected areas and wetlands. Map adapted from Sinkala et al., 2014 [68].

Zambia is a landlocked country covering 752, 610 km^2^ between latitudes 8° and 18° south of the Equator and longitudes 22° and 34° east of the Greenwich Meridian (Figure 1). The country shares borders with eight Southern Africa Development Community (SADC) member countries [13] and is composed of 10 administrative areas called provinces. Zambia is regarded as one of the least urbanised countries in Africa with an estimated total population of 13.4 million people, majority of whom (61%) are concentrated mainly in the rural areas [14]. Agriculture contributes about 21.5% to gross domestic product (GDP), out of which 28% is from the livestock subsector [15, 16]. The national per capital income is one of the lowest in the world estimated at US$ 1,700 for 2012 [17]. Of the total land, 47.5% is agricultural land, and 85% of this agriculture land is under pasture, signifying the availability of more land for grazing than arable [2, 18]. The remaining 52.5% is forest, national parks, and game management areas [19]. The livestock production system can broadly be categorised into traditional and commerial sectors [20].

### 2.1. Traditional Livestock Production Systems

Traditional farmers occupy the communal areas owning 84% of the estimated 3.4 million cattle, 68% of the 1000 sheep, 97% of the 1 million goats, and 90% of the 1.5 million pigs [1, 21]. Cattle are raised on communal land under an extensive grazing system. In the midlands and southern part of Zambia, three linked herding patterns exist: village resident herds (always in the villages), transhumant herds (move between village and the floodplains), and interface herds (permanently stay on the floodplains) [22, 23]. In the rest of the country, cattle are grazed in the communal areas as village resident herds and the distances covered are dependent on season and availability of pasture [20]. The predominant cattle breeds are Zebu and Sanga [20].

#### 2.1.1. Communal Landuse Systems

As pointed out above, a large proportion of livestock is owned by traditional farmers who mainly occupy communal lands. These farmers share grazing pasture and watering points. This farming system allows for cattle from different owners to share grazing pastures and water sources enabling disease transmission between different herds. During the dry season, the water sources become scarce and grazing pastures shrink into a few areas that are close to water sources and as such this tends to increase the animal population density at the water sources and grazing pastures. Generally, it is known that transmission of FMD is exacerbated by increase in population density enhancing the transmission of the disease through contact between the infected and noninfected animals [24]. Besides, given that cattle have been shown to carry their virus from last outbreaks for 2 to 3.5 years [25, 26], environmental stress due to decrease of grazing pastures and overcrowding at water sources may cause recrudescence of the disease in the carrier animals which could lead to occurrence of outbreaks. This scenario is likely to be the cause of outbreaks in the Northern region in between Mbala and Nakonde where there is no transhumance grazing system practiced and yet farmer share grazing pastures and water sources.

#### 2.1.2. Transhumance Grazing Systems

The midlands and southern part of Zambia practice a transhumance grazing system. This poses a significant challenge because of the large population densities of cattle that share grazing pastures during the dry season in the Kafue basin and on the wetland areas of the Zambezi River in the areas between Kazungula and Sesheke districts. These areas tend to have the highest recurrence of outbreaks and in most cases outbreaks occur during the period of increased population densities of cattle in the dry season. The major challenge with the transhumance grazing system in the Kafue Flats being the most widely affected area is that cattle come from the upland areas into the wetland areas for grazing. The area has been estimated to account for >500,000 cattle herds that graze in the wetland areas [9]. This allows for increased contact thereby increasing the possibility of transmission from infected to noninfected animals. At the end of the dry season, animals are moved back from the wetlands to the upland areas during which time they spread the outbreak further [9, 27]. Generally, this area makes it difficult to implement movement restriction and the cattle herds at risk are usually many and as such ring vaccination is difficult to implement. Put together, several factors make this area highly vulnerable to FMD outbreaks and at the same time difficult to put timely control measures in place.

### 2.2. Commercial Farming Systems

Commercial farms keeping mostly exotic breeds of beef and dairy cattle are run as private business enterprise characterised by high production efficiency and high offtake rates to the market. Commercial farms are concentrated along the line of rail within the midlands and southern parts of the country and are the largest supplier of milk and beef to the urban population [28]. Occassionally, disease outbreaks from the traditional sector might spill into these farms. Outbreaks of FMD in the sector causes huge losses in productivity and income from movement bans which in turn affects the national economy through drop in supply and general rise in prices of beef and other competing commodities [29].

### 2.3. Beef and Dairy Products Market Forces

One of the greatest challenges faced by the Zambian government in the control of FMD is that the market forces for beef and dairy products are placed in Lusaka and the Copperbelt provinces were the human population density is high and yet the largest producing areas of beef are in the communal lands and transhumance grazing areas of the central, southern, and western provinces of the country where the recurrence of FMD outbreaks is high. And as such, during outbreaks, there is a tendency to move animals illegally to abattoirs and slaughter slab in the urban areas. These illegal movements exacerbate the transmission of FMD. Enforcing movement restrictions is often a challenge and disease easily moves to new areas rapidly before it can be put under control.

### 2.4. Transboundary Transmission

Transborder transmission of FMD between cattle is a major problem in Southern Africa. For example, the Northern Region of Zambia covering the area between Mbala and Nakonde bordering the Rukwa and Mbeya regions of Southern Tanzania tends to share the same serotypes of FMD and outbreaks in this area tend to occur at the same time suggesting the likelihood of transmission of the disease between the two countries [30–32]. Similarly, outbreaks of FMD between Zambia, Zimbabwe, Botswana, and Namibia in the south tend to follow transborder transmission across the Zambezi River. Similarly, FMD outbreaks have been reported across the border between Zambia and Malawi in the east [33]. The major challenge with transboundary transmission of FMD is that surveillance systems are not synchronized between countries and during outbreaks, control strategies are not carried out simultaneously. Hence, this raises the risk of back-and-forth transmission of FMD across borders given the lack of synchronized control and surveillance strategies.

## 3. Challenges in the Wildlife Production Systems

The Zambia Wildlife Authority (ZAWA) is a government institution responsible for the management of wildlife through protected areas and game ranches [34].

### 3.1. Game Management Areas

The protected areas comprise 19 national parks (NPs) and 35 game managements areas (GMAs). The GMAs act as buffer zones between NPs and human settlement areas (Figure 1). This buffer zone otherwise known as human/livestock/wildife interface is defined as a mosaic of human settlement, livestock grazing, wildlife conservation, and cultivation [35]. Legal hunting is permitted in the GMAs while NPs are reserved for conservation and education purposes. Several studies have shown how coexistence of wildlife with livestock in the GMAs affects transmission of different wildlife diseases between livestock and wildlife [27, 36–39]. Hence, it is likely that GMAs which are most located in the wetlands such as the Kafue basin where transhumant and floodplain herds share grazing pastures with wildlife serve as interface area for disease transmission between wildlife and livestock [9, 24, 27]. Hence, this would acount for reasons why FMD outbreaks are the highest in the Kafue flats where cattle and buffalo share common grazing pastures.

### 3.2. FMD Wildlife Reservoir

The African wild buffalo, present in most NPs, is a major tourist attractions contributing over 12.4% of the total hunting revenue through safari hunting for game trophies, ecotourism, and photographic tourism [37]. The NPs and GMAs are not fenced and human encroachment into wildlife areas in search of pasture and water is common. Unfortunately, the African buffalo as described earlier is known to be a reservoir for FMDV and its interaction with domestic animals at the interface areas or GMAs poses a significant danger on resurgence of FMDV outbreaks. The African buffalo is an attractive collection for game ranchers. This would account for the reason why the African buffalo is not commonly reared on game ranches because FMD has proved to be constraint in rearing the African buffalo [34, 37, 40, 41]. Generally, it is becoming common practice that all ungulates captured from national parks and GMAs known to be endemic with FMD are screened of FMD to ensure they do not carry the disease to game ranches which are located in areas close to livestock production areas. Consequently, this makes FMD a constraint to the expansion of the game ranching industry because of the prohibitive capture operation and FMD screening costs [34, 37, 41].

### 3.3. Expansion of the Game Ranching Industry

Game ranches have emerged since 1978 as an alternative conservation strategy to conserve the depleting wildlife resources [42]. Game ranching is considered environmentally sustainable and economically viable industry because of its ability to integrate ecotourism and wildlife utilization with livestock keeping [37], while in those few game ranches with buffalo populations, the FMD status remains unknown [43].

## 4. FMD in Zambian Livestock and Wildlife

The SAT FMD is known to be maintained by the wild buffalo, which is the stable biological reservoir of the FMDV [44]. However, transmission from buffalo to cattle is not adequately understood because of little published research on the subject [45, 46]. Elsewhere transmission of FMD from buffalo to cattle has been demonstrated under both experimental and natural conditions although it still remains a rare event [47–50]. In addition, other wildlife species have been described to play an intermediary role in different ecological systems [49, 51, 52]. Attempts to transmit FMDV from persistently infected cattle under controlled field conditions have not yielded success except the one recorded in Zimbabwe [47].

The role of small stock (goat and sheep) in the transmission of FMD is regularly debated but according to Hunter [53], evidence exists to indicate that small stock plays no role as carrier animals. As a result, FMD had been controlled in several countries without vaccinating small stock [53]. In Zambia, few reports of FMDV cases in small stock exist partly because of subclinical manifestation of the disease in these species, low numbers of goats and sheep, and lack of surveillance efforts targeting small livestock [54, 55]. However, recent outbreaks of FMDV in Botswana involving small livestock indicate that the disease in this host species still remains significant [56].

## 5. Challenges of Controlling FMD in Zambia

Methods of control used in Zambia from 1933 to 1980s have been summarized [55]. The methods of FMD control that are being implemented include surveillance, quarantine, movement control, and biannual vaccination of cattle in the high risk areas.

### 5.1. Aphthization

This practice was used from the 1940s until 1973 when it was abolished [57]. Aphthization involved deliberate infection of cattle with infectious materials obtained from neighbouring infected farms [58]. The method was introduced as a means to speed up infection in cattle population by burning out the disease. Once all animals were exposed, it was assumed that they would develop immune protection from natural exposure.

The limitations of aphthization have been described [55]. It is probable that aphthization intensified the virulence of the FMDV and created a reservoir of infection in wildlife and other livestock for retransmission to cattle as their immunity wanes [55, 59]. Verification of this theory has not been possible because the central role of wildlife, mainly buffalo in FMD transmission, was only identified in the 1960s as a result of operation Noah [60]. However, given growing evidence that cattle exposed to FMD can serve as short term reservoirs with virus lasting up to 3.5 years in the oral-pharyngeal area suggests that aphthization would cause a danger of increase in the number of carriers in populations exposed to FMD and as such this strategy of FMD control was abandoned.

### 5.2. Vaccination

From the 1960 onwards, vaccination was introduced using an attenuated SAT 2 vaccine in villages surrounding an outbreak area while for infected villages aphthization was used until its later was abolishment [57]. In 1973, an inactivated adjuvant vaccine replaced the live attenuated vaccine [57]. Vaccination has traditionally been done in cattle except once in 1979 in Livingstone when other species like goats, pigs, and sheep were vaccinated with T155 bivalent SAT1 and SAT 2 vaccine supplied by Wellcome (Kenya) limited [61]. The source of vaccines remained Wellcome laboratories of Kenya until early 1990s when the contract was awarded to Botswana Vaccine Institute.

#### 5.2.1. Private Sector Involvement

In the late 1990s and early 2000s, vaccinations were done through contracts to private veterinarian [33]. These initiatives were targeted to support the privatization of veterinary services and improve service delivery. However, vaccination coverage during these contracts was generally unsatisfactory due to the many problems associated with awarding of these contracts to the private sector (Peter Sinyangwe, personal communication).

#### 5.2.2. Vaccines in Use

The vaccines currently in use consist of complete, chemically inactivated 146S virion particles combined with saponin-alhydrogel adjuvant [25]. The vaccines are multivalent that provide protection against multiple serotypes and a potency of at least 3 PD_50_ per dose [3] but this is still a challenge for Zambia with five serotypes and multiple topotypes that have to be incorporated into a single vaccine. The vaccination does not induce sterile immunity and animals may still be able to infect nonvaccinated animals and may also become persistently infected [25, 26]. As such, the urgent need for new SAT vaccine strains with good immunogenicity for use in Africa was highlighted at the recent Global FMD Research Alliance (GFRA) congress [62].

#### 5.2.3. Vaccination Strategy

The inactivated vaccines in use induce short-lived immunity and require a booster dose every 4–6 months [53]. However, some areas are inaccessible due to annual flooding in some of the FMD high risk areas. Further, the lack of reliable census figures in the traditional sector makes verification of the required 80% vaccine coverage problematic [25]. Whilst vaccination of cattle with viruses that are antigenically closely related to those carried by buffalo form an integral part of FMD control in Southern Africa [53, 63], it has not been a common practice in Zambia [64]. This is because collecting samples from buffalo and having them tested at reference laboratories is expensive. Usually during vaccinations following an outbreak, no distinction is made between infected and susceptible animals. Strains used for routine vaccinations are presumed effective during vaccination response to outbreaks. However, there is no empirical data that show the efficacy of these vaccines in the various situations they have been applied.

#### 5.2.4. Inadequate Vaccine Storage Facilities

Inadequate cold-chain facilities have been a major challenge in handling the vaccine in the field. This is particularly the case during the second round of vaccination around October to November when temperatures sometimes exceed 35°C. Frequent breakdowns of the cold chain have to be suspected, leading to degradation of the vaccine antigen (146S particles) into 12S particles which elicit almost no antibodies. However, up to now, there are no laboratory data to confirm this hypothesis [65]. Given the status quo, Zambia would benefit from vaccines with improved stability which are less reliant on a cold chain.

### 5.3. Movement Control

#### 5.3.1. Livestock Movement Control

Challenges exist in controlling movement in the traditional sector due to communal grazing, transhumance, use of oxen for transport, hiring of bulls for mating, and other vices like shifting from village to village; mafisa and lobola [66]. The immunological diversity in prevalent serotypes and topotypes that exist in Zambia may render FMD difficult to control in present circumstances [67, 68].

Movement restrictions since the first outbreak in 1933 have generally not been satisfactory in Zambia [55]. After the outbreaks recorded in 1934, 1953, 1973, 1980, 2004, and 2007 geographical spread to other areas was recorded. For example, during the 2007 Sesheke outbreak, the disease spread eastwards to Kazungula, northwest to Senanga, Mongu, Kalabo, and Shangombo near the border with Angola. It is suspected that following the closure of the local abattoirs imposed by the veterinary officials in enforcing the total movement ban, farmers opted to search for market elsewhere spreading the disease in the process. The situation was exacerbated by the fact that oxen are a common means of transport in the Barotse sands (an extension of the Kalahari sands), where motor vehicle transport is not often used because of poor traction afforded by the sandy terrain.

#### 5.3.2. Fencing Separating Wildlife from Livestock

Separation of wildlife from livestock using game fences is not currently practiced in Zambia because of the prohibitive costs. This is despite movement of wildlife and livestock within and between ecosystems including transborder movement being rife. Previously, game fences were erected in 1950s to control movement of tsetse flies from wildlife to cattle around the Kafue national park until the 1980s when they collapsed because they could not be maintained [66]. The effectiveness of these fences in controlling FMD is questionable given that outbreaks were recorded during the period. Since the 1990s, movement of livestock and wildlife (ungulates) from high risk areas is not permitted without prior screening for FMD. The screening is through serology and virus isolation using probing samples collected from the oral pharyngeal area while serology is by nonstructural protein (NSP) ELISA and liquid phase blocking ELISA (LPBE).

### 5.4. FMD Diagnostic Capacity

Diagnosis of FMD is based on clinical suspicion followed by serology and virus isolation.

#### 5.4.1. Serology

Serology using NSP and LPB ELISA is undertaken locally at Central Veterinary Research Institute (CVRI). The world reference laboratory protocol for LPBE has been adopted at CVRI with technical support from the regional reference laboratories of Onderstepoort Veterinary Institute (OVI) of South Africa and Botswana Vaccine Institute (BVI) in Botswana [69] through the initiatives of the laboratory subcommittee of the Livestock Technical Committee of SADC. Briefly, the NSP detects antibodies to the polyproteins 3ABC of FMDV as an indicator of past or present infection for any of the five serotypes of the virus existing in Zambia, whether or not the animal has been vaccinated [70]. The LPBE on the other hand detects antibodies to viral structural proteins, is more serotype specific and highly sensitive, and detects antibodies elicited by both vaccination and infection. The LPBE is a prescribed test for trade because it is appropriate for confirming previous or on-going infection in nonvaccinated animals as well as for monitoring the immunity conferred by vaccination in the field if purified vaccines have been used [71, 72].

The NSP and LPB ELISAs being used at CVRI require to be optimised for the FMDV serotypes circulating in Zambia. The serology could be improved by validating the NSP assays that are being used to develop confidence in the results. In order to deal with ambiguity, the LPBE test results require investment in virus neutralisation test (VNT). The VNT could be used as confirmatory test in the detection of FMDV-specific antibody in animals previously exposed to the virus [73, 74]. The VNT requires technical skill to be performed accurately and is dependent on cell culture facilities [6], which may not be conducive for laboratories in endemic regions. The vaccines currently in use in Zambia are not purified and the repeated vaccinations that exist may lead to NSP being produced that may be confused with NSP from natural infection. This is a challenge in the interpretation of NSP test results and impacts on the ability to export from FMD-controlled regions. Currently, it is difficult to differentiate between vaccinated and previously infected animals in Zambia where nonpurified vaccines are used [75].

#### 5.4.2. Virus Isolation

Samples for virus isolation and characterisation are routinely sent to regional reference laboratories of OVI, BVI, and World Reference Laboratory (WRL) in the United Kingdom, because Zambia lacks biosecurity level 3 (BSL3) facilities. However, this involves the transportation of samples containing infectious FMDV which represents a significant biosecurity hazard and also incurs significant costs. The success of virus isolation is dependent on the sample quality and requires special transport conditions from the sampling point to the laboratory [75]; this presents challenges for Zambia with poor laboratory infrastructure and trained manpower.

Samples sent to reference laboratories are for virus isolation, antigen ELISA, and sequencing and phylogenetic analysis [75]. Such samples may include vesicular tissues and fluids from outbreaks and oropharyngeal scrapping for surveillance of carriers because more than 50% of infected animals may become carriers [26, 76] but the duration of persistence and proportion that become carriers in Zambia are not known. It is not clear whether these carriers transmit infection to susceptible cattle [75]. Sequencing of the VP1 gene or the full genome provides forensic evidence for the source of the FMDV in outbreak situation as well as in tracking the movement of buffalo strains [77].

#### 5.4.3. Field Diagnostic Tests

The main challenge facing FMD diagnostic capacity in Zambia is unavailability of real time on the spot field diagnostic tests that could be used during outbreaks to identify circulating FMDV serotypes and topotypes for quick vaccine matching. To overcome this challenge, several tests have been developed.

(*1) Lateral Flow Devices.* Lateral flow devices (LFDs) have been developed and evaluated which are either serotype specific [78] or can detect all seven FMDV serotypes [79, 80]. LFDs are immunochromatographic tests that allow the diagnosis of FMDV at the site of a suspected outbreak. The LFD can utilise vesicular fluid or vesicular epithelial suspensions but not nasal swabs or sera [78]. The method makes use of capture and detection of monoclonal antibodies or specific polyclonal antisera on a strip test and studies done this far have shown the test to be as sensitive and specific as the antigen ELISA; however, the sensitivity of the strip test may differ for the various FMDV strains [81].

(*2) Portable Real-Time PCR Platforms.* Portable real-time PCR platforms offer many advantages in endemic countries like Zambia [82]. An example of is the Enigma FL field laboratory platform (Enigma diagnostics) capable of nucleic acid extraction, PCR thermocycling, and analysis of data without the requirement for user intervention and has been tested for FMD diagnosis [82]. These platforms require to be evaluated whether field-based assays can detect new viruses as they continue to evolve in sub-Saharan Africa. These platforms can be utilized by nonspecialists and are designed to perform all the steps of a RT-PCR test, such as nucleic acid extraction and performing RT-PCR which is valuable for evaluating carriers.

(*3) Loop-Mediated Isothermal Amplification.* The loop-mediated isothermal amplification (LAMP) assay is an alternative molecular detection technique with similar performance to real-time reverse transcriptase polymerase chain reaction (rt RT-PCR) that has been used widely for the detection of RNA and DNA viruses that infect livestock [83]. LAMP has the capacity to identify on the spot serotypes and carriers in the field. Yamazaki et al. [84] developed a multiplex RT-LAMP approach to accommodate the high sequence variability encountered in RNA virus genomes particularly for SAT strains and found the analytical sensitivity to be comparable to the singleplex RT-LAMP assays [83].

*(4) Novel Diagnostic Assays.* Additional novel diagnostic assays such as biosensors [85], microarrays [86], gold nanoparticle (GNP) improved immuno-PCR (GNP-IPCR**) **[87], and nucleic acid sequence based amplification (NASBA) [88] have shown promising ability for rapid and reliable diagnosis, surveillance screening, and strain typing for FMDV for sub-Saharan Africa.Some of the limitations of these assays are that they are yet to beoptimised for the FMDV SAT serotypes with a high degree of sequence variability like Zambia. The costs involved per test will also determine how widely these assays will be used especially for developing countries like Zambia. The field-based novel assays will require training of personnel and some laboratory-based tests require specialised equipment which is not readily available, together with personnel capable of correctly interpreting and analysing the datasets produced.

### 5.5. Emergency Preparedness

FMD disease outbreaks require immediate and defined response in case of an incursion. Emergency response plans exist in document form but require translation into action such as simulation exercises. Although an emergency disease control fund has been created, funding is usually inadequate and contingency vaccine banks have not been established and, as such, there is a long time lag between disease identification and institution of control measures [1, 43]. This status quo usually exacerbates the spread of the disease resulting in increased cost of control [4].

### 5.6. Sociological Aspects

Understanding smallholder farmers knowledge, attitudes, and perception and how they adapt to FMD outbreaks is inadequate [29]. During the 2004 Namwala outbreak, farmers abandoned their usual* Lutanga* (local name for transhumant village) located in the epicentre of the outbreak. For fear of the disease, they opted for new grazing areas further away from the epicentre and consequently spreading the disease along the way.

### 5.7. Delivery of Veterinary Services

Zambia has experienced a decline in delivery of veterinary services since privatization of veterinary services in the early 1990s and the introduction of the structural adjustment programmes [66]. This may have negatively affected the surveillance required for early warning system, disease reporting, and confirmation as required for the FMD progressive control pathway [89, 90].

## 6. Impact of FMD on the Zambian Economy 

Impacts of FMD in sub-Saharan Africa have been described in several studies but none for Zambia [29, 91]. Experience in developed countries has shown that it is impossible to economically do livestock farming in the presence of FMD [92]. The impacts reported here were based on Knight-Jones and Rushton [93] framework.

### 6.1. Direct Visible Losses

Direct losses limit livestock productivity creating food insecurity and contributing to malnutrition. During the 2004 Kafue flats Namwala outbreak, the calving rate reduced during the calving season as most cows did not conceive (Musso Munyeme, personal communication). Herds' men had earlier observed that the bulls were failing to mount the cows during heat periods because of sores on the feet. On average around 300,000 calves are born every year in this region [94]. During the 2008 outbreak of FMD at a commercial farm in Mazabuka district, milk production was reported to have dropped from 25.5 to 0.5 litres per cow per day (Martin Ndhlovu, personal communication). In Zambia, traditional farming using draught power accounts for the largest production of crops [95]. Elsewhere oxen have been observed to stay off plough for the whole season when outbreaks occur in a cropping season with drop in draught power of 60–70% after one month following infection [25].

### 6.2. Direct Invisible Losses

Low conception rates of 52 to 69%, calving rates averaging 40 to 58%, and long calving to conception intervals of 18 to 20 months characterize the reproductive efficiency in Zambia [43]. This may be a result of endemic FMD infections [29]. Fertility reduces because of increase in abortion rates of up to 10% [25] and prolonged intercalving interval by 12 months due to delays in conception [96].

### 6.3. Indirect Impacts

It is estimated that the ZambianGovernment spends over US$ 2.7 million yearly on procuring vaccines and conducting and monitoring vaccination campaigns [97]. Biannual vaccination campaigns conducted each year consume the productive time of both the farmer and the field extensive officers to invest in other productive activities. This is difficult to quantify but in part offers a probable explanation to the slow growth in the livestock sector [29]. With respect to government cost of control, it is estimated that the cost of erecting and maintaining one checkpoint during the 2004 Namwala outbreak was US$ 10,000 for two weeks [43]. This figure may rise if the period is longer and sometimes three or more checkpoints were required. The cost of surveillance, extension, and farmer training in disease identification and awareness adds to expenditure.

### 6.4. Internal Market Constraints

In Zambia, demand for beef is the highest in Lusaka with a human population of 2,196,996 and the Copperbelt with 1,958,623 [14]. During disease outbreaks, movement restrictions imposed on livestock from affected regions create a deficit that leads to upswing of prices of beef and competing goods [29]. Zambia currently imports beef to satisfy local market demand and any drop in the local source worsens the situation. Another consequence of being a country with endemic FMD is that Zambia cannot participate in international livestock trade. For example, as a result of the 2010 Mbala serotype O outbreak, Botswana imposed a total ban on import of maize bran from Zambia. Zambia exports in excess of 30,000 metric tons of maize bran annually to Botswana [98]. It is further estimated that over US$ 3 Million was lost in revenue during the ban (George Phiri, Grain Traders Association, personal communication). In 2008, over 500 sable antelopes (*Hippotragus niger*) could not be exported to South Africa because of trade politics related to FMD. Losses in income from exports of these prime sable antelopes have been estimated at US$ 35 million annually [99]. The loss in income from potential exports of beef and dairy products if a disease free zone existed has been estimated to be over US$ 1.6 billion per annum [2].

### 6.5. Effect on the Internal Economic Growth

The period between 2000 and 2010 was characterised by increase in the numbers of FMD outbreaks in comparison to previous decades. During the same period, the Zambian human population was increasing at an average of 2.8% per annum [14]. The cattle decline experienced in the early 2000s was probably due to droughts and eventual FMD outbreaks on the Kafue flats. The reduction in calving and conception rates alluded to earlier may have contributed. Even though the country recorded an upswing in cattle numbers from 2,381,421 in 2005 to 2,559, 953 in 2010 [100], the growth was not commensurate with the human population growth. Probably the extension of the 2004 Namwala outbreak to 2005 and 2006 as well as the 2007 to 2009 Mwandi outbreak in the middle Zambezi basin may have contributed. Zambia also experienced positive economic growth in real GDP from 2000 to 2010 [15] but the economic growth was slow to support the population growth. This was mainly because the growth was driven by improved performance in mining and construction sectors while agriculture upon which 80% of the population depends did not perform well, recording relatively low average growth rates, inadequate infrastructure, and poor market access [1, 101].

## 7. Discussion and Recommendations 

The challenges of controlling FMD and economic impacts of its occurrence have been highlighted. Zambia has developed a risk based national FMD control strategy but the implementation process needs to be scaled up. In order to maintain Zambia's level 2 PCP status, more work is needed to improve the organisation of infrastructure including the diagnostic capacity, the epidemiology capacity to provide evidence on the absence of disease, and if present the magnitude and mitigation measures being implemented. Zambia must undertake an annual assessment of evidence based on FMD related activities being undertaken to understand the epidemiology and control the disease [75]. Understanding the local epidemiology of FMD and the active monitoring of the virus circulating are the foundations of the PCP-FMD and activities to meet the requirements [75]. Further determination of the factors influencing the maintenance and spread of the disease as well as knowledge of circulating subtypes of FMDV is essential for effective control of the disease. At present, knowledge gaps exist in determinants of disease occurrence, mechanisms of virus maintenance, and transmission.

Control measures appear to be ineffective to stop the reoccurrence of disease [11]. FMD control requires huge financial investments in infrastructure and research as there are many epidemiological factors needing clarification [102]. There is little research done on FMD in Zambia in comparison to other southern African countries.

Therefore, FMD control in Zambia currently must focus on risk reduction measures for specified commodities rather than country or at least zonal freedom [103]. One alternative for sustainable FMD control under the existing endemic situation could be Article 8.5.25 of the Terrestrial Animal Health Code [104] adopted by the Southern African Development Community Livestock Technical Group [105]. Commodity based trade (CBT) standard [104] offers a cost effective alternative in deriving benefits from livestock and wildlife conservation in endemic settings in comparison to zoning. The CBT may be utilized in the short to medium term to accrue benefits whilst implementing the PCP in the long term. Care must be made to assess the risks of other diseases such as tuberculosis and brucellosis among others [106]. CBT and the PCP need to complement each other for Zambia to accrue the benefits from livestock and wildlife trade. The adoption of CBT is envisaged to boost intraregional trade that may accelerate sub-Saharan Africa's economic growth. Therefore, guidelines to operationalize CBT standards when available may offer countries like Zambia an alternative for FMD control in view of the TFCA that have been created.

FMD is essentially a disease of poverty in Zambia because of the negative effects on livestock production and productivity, competitiveness and access to lucrative international markets. The disease is a drain on the national treasury in a country where resources are scarce and FMD control competes with other priority sectors like health and education. Even within agriculture, priority is given to maize and fertilizer support programs, the commodity being a staple food and preference is understandably for economic and political reasons.

Clearly, FMD impacts negatively on the Zambian economy and may be responsible for the low economic growth [29]. Zambia's FMD endemic situation inhibits delivery of economic benefits through animal exports with 80% of its population being dependent on agriculture [1]. The FMD endemicity may be the reason for poor investment in livestock as noted from the stagnant cattle numbers in relation to the upswing in human population. For example, whilst the human population has been increasing annually at 2.8%, the cattle numbers have relatively remained stagnant [14, 21].

In order to ensure effective disease surveillance and monitoring, there is need to invest in a low containment biosecurity level 2 laboratories for the country to engage in monitoring of FMDV strains circulating in livestock and wildlife. Investment in diagnostic tests may need scaling up to monitor field performance of vaccines and determine serological levels needed for protection. Included is the monitoring of the development of carriers, an important aspect for regulating movement control and creation of disease freedom. Budget allocation for diagnostic kits and in-house reagents with proficiency testing with regional centers must be scaled up. Onsite tests that can identify incubating animals not fit for vaccination may need to be developed. We propose an annual review of the vaccination programs using empirical data from carrier development, vaccine efficacy, and coverage surveys. The review may also include social economic factors that relate to vaccination and uptake. Therefore, to understand the epidemiology of FMD endemicity in Zambia, various research approaches may be required. Included is the identification of risk determinants, including sociological, epidemiological, and ecological factors, in order to be able to advance on the FAO/OIE progressive control pathway for FMD.
